# Supplementary material for: Coronavirus-Related Health Literacy: A Cross-Sectional Study in Adults during the COVID-19 Infodemic in Germany
Source: Int J Environ Res Public Health. 2020 Jul 30;17(15):5503. doi: 10.3390/ijerph17155503 (PMC7432052; doi:10.3390/ijerph17155503)
Supplement: Supplementary file 1 [file ijerph-17-05503-s001.pdf]

Supplement 1

Table S1. Correlations between health literacy items (HLS-COVID-22) and sociodemographic variables.

	Health Literacy Items and Mean Score	Sex		Age		Education		Household Income		Children <18 Years		Region	
		ρ	p	ρ	p	ρ	p	ρ	p	ρ	p	ρ	p
**Item**	Health literacy mean score	−0.019	0.532	−0.010	0.758	0.072 *	0.020	0.048	0.122	−0.006	0.845	−0.013	0.671
1	find information about the coronavirus on the internet?	−0.013	0.686	−0.059	0.058	0.106 **	0.001	0.068 *	0.028	−0.042	0.174	−0.040	0.202
2	find information on the internet about protective behaviors that can help to prevent infection with the coronavirus?	0.020	0.511	−0.033	0.284	0.113 **	0.000	0.055	0.076	0.010	0.746	−0.001	0.983
3	find information in newspapers0. magazines and on TV about behaviors that can help to prevent infection with the coronavirus?	0.012	0.711	0.078 *	0.011	0.032	0.310	0.029	0.351	−0.003	0.930	−0.047	0.128
4	find information on how to recognize if I have likely become infected with the coronavirus?	−0.004	0.898	−0.009	0.784	0.027	0.387	0.006	0.850	0.028	0.368	0.036	0.249
5	find information on how to find professional help in case of coronavirus infection?	−0.025	0.414	0.009	0.773	0.038	0.224	0.012	0.695	0.016	0.611	0.014	0.658
6	find information on how I much I am at risk for being infected with the coronavirus?	−0.051	0.104	−0.003	0.911	0.011	0.733	−0.026	0.406	−0.003	0.922	−0.030	0.336
7	understand your doctor\`s0. pharmacist\`s or nurse\`s instructions on protective measures against coronavirus infection?	0.011	0.719	0.000	0.993	0.098 **	0.002	0.047	0.127	0.010	0.741	−0.029	0.356
8	understand recommendations of authorities regarding protective measures against coronavirus infection?	0.038	0.217	−0.002	0.943	0.054	0.085	0.045	0.145	−0.019	0.540	−0.031	0.323
9	understand advice from family members or friends regarding protective measures against coronavirus infection?	0.039	0.204	−0.036	0.248	0.102 **	0.001	0.030	0.334	0.000	0.998	−0.007	0.833
10	understand information in the media on how to protect myself against coronavirus infection?	0.046	0.140	−0.018	0.561	0.060	0.053	0.032	0.307	−0.051	0.099	−0.056	0.070
11	understand risks of the coronavirus that I find on the internet?	0.008	0.797	−0.035	0.256	0.058	0.064	0.050	0.111	−0.036	0.242	0.004	0.898
12	understand risks of the coronavirus that I find in newspapers0. magazines or on TV?	−0.005	0.862	0.025	0.419	0.041	0.189	0.034	0.267	−0.023	0.463	−0.011	0.727
13	judge if information on the coronavirus and the coronavirus epidemic in the media is reliable?	−0.068 *	0.028	−0.040	0.203	0.002	0.939	0.029	0.343	0.042	0.177	−0.027	0.379
14	judge which behaviors are associated with a higher risk of coronavirus infection?	−0.046	0.143	−0.042	0.172	0.099 **	0.001	0.050	0.109	−0.010	0.746	−0.035	0.263
15	judge what protective measures you can apply to prevent a coronavirus infection?	−0.029	0.351	−0.033	0.289	0.068 *	0.028	0.041	0.189	0.002	0.951	−0.013	0.671
16	judge how much I am at risk for a coronavirus infection?	−0.033	0.287	0.010	0.737	0.034	0.275	−0.012	0.699	−0.026	0.408	0.025	0.417
17	judge if I have been infected with coronavirus?	−0.077 *	0.013	0.026	0.397	−0.016	0.606	−0.014	0.652	0.007	0.829	0.000	0.993
18	decide how you can protect yourself from coronavirus infection based on information in the media?	0.024	0.447	−0.026	0.397	0.055	0.075	0.026	0.397	0.005	0.878	−0.023	0.460
19	follow instructions from your doctor or pharmacist regarding how to handle the coronavirus situation?	0.012	0.690	0.041	0.187	0.080 *	0.010	0.031	0.316	−0.025	0.430	0.008	0.807
20	use information the doctor gives you to decide how to handle an infection with the coronavirus?	−0.042	0.178	−0.029	0.348	0.037	0.235	0.033	0.290	0.002	0.950	0.025	0.420
21	use media information to decide how to handle an infection with the coronavirus?	−0.023	0.453	−0.028	0.376	−0.006	0.857	0.019	0.548	0.007	0.813	0.024	0.438
22	to behave in a way to avoid infecting others?	−0.008	0.805	0.062 *	0.047	0.015	0.623	0.048	0.121	−0.077 *	0.013	−0.018	0.558

N = 1037; Spearman coefficient (ϱ); p-value (p); ** = $p < 0.01$ (two-tailed); * = $p < 0.05$ (two-tailed); To transfer mean scores to frequently reported metric ranging from 0–500. this formula can be used: y = (mean − 1) × (50 ÷ 3). The inverse formula0. y = (3 × mean ÷ 50) + 10. transforms scores on the transformed metric to mean scores on the original response format (1–4).

.

Table S2. Correlation between information variables and sociodemographic indicators.

		How Well Are You Informed about Coronavirus?	Do You Feel Confused about Coronavirus Information?	Sex	Age	Education	Household Income	Children <18 Years	Region
How well are you informed about coronavirus?	ϱ	10.000	−0.224 **	−0.003	0.039	0.026	0.068 *	−0.001	−0.061
	p		0.000	0.931	0.215	0.404	0.028	0.976	0.051
Do you feel confused about coronavirus information?	ϱ	−0.224 **	10.000	0.085 **	−0.112 ***	−0.049	−0.003	0.112 ***	0.020
	p	0.000		0.006	0.000	0.115	0.916	0.000	0.517

N = 1037; Spearman coefficient (ϱ); *p*-value (*p*); *** = p < 0.001; ** = p < 0.01; * = p < 0.05.
